# Exploring the Nexus Between Fertility Rates and Geopolitical Risk with Intelligence Methods: A Multifaceted Analysis

**DOI:** 10.3390/healthcare12222205

**Published:** 2024-11-05

**Authors:** Maria Tzitiridou-Chatzopoulou, Georgia Zournatzidou, Ioannis Tsakiridis, Christos Tsakalidis

**Affiliations:** 1 Midwifery Department, School of Healthcare Sciences, University of Western Macedonia, Koila, 50 100 Kozani, Greece; mtzitiridou@uowm.gr; 2 Department of Business Administration, University of Western Macedonia, 51 100 Grevena, Greece; 3 Department of Accounting and Finance, Hellenic Mediterranean University, 71 410 Heraklion, Greece; 4 Third Department of Obstetrics and Gynecology, School of Medicine, Aristotle University of Thessaloniki, 54 124 Thessaloniki, Greece; igtsakir@auth.gr; 5 Second Neonatal Department, Neonatal Intensive Care Unit, Aristotle University of Thessaloniki, “Papageorgiou” General Hospital of Thessaloniki, 564 29 Thessaloniki, Greece; golema@auth.gr

**Keywords:** geopolitical risk, predictability, birth rate data, disruptions, machine learning, fertility rate, demographic challenges

## Abstract

**Background/Objectives**: This paper presents an analysis of birth rate statistics, specifically focusing on recorded births in Scotland. The main research objective focuses on investigating the influence of geopolitical concerns on birth rate forecasts. Specifically, we examine whether individuals may choose to postpone or abstain from having children during times of conflict or political turmoil due to concerns about personal safety, the welfare of their children, or uncertainty about the future caused by geopolitical risks. Additionally, this study examines how disruptions to healthcare services, such as limited access to prenatal care and maternal health facilities, can affect birth outcomes and lead to changes in birth rates. **Methods**: To approach the research objective both machine learning algorithms and classical statistical procedures. Also, as part of the current analysis, the Geopolitical Risk Index has been applied as an extra factor to predict the birth rate. **Results**: The results of our study demonstrate the effectiveness of machine learning in producing precise predictions in this field, while emphasizing the significant influence of geopolitical risk on comprehending the dynamics of birth rates in Scotland. **Conclusions**: This study examines the effectiveness of several machine learning regression models in accurately predicting the number of births in Scotland using data that is not included in the model training process. Findings show promising outcomes in predicting births, while geopolitical instability has been indicated as a substantial influence on birth rates and fertility rates.

## 1. Introduction

This study highlights the intricate nature of human fertility, which is influenced by a combination of biological, social, and economic elements. Factors such as education levels, work prospects for women, childcare expenses, and larger economic factors all contribute to determining decisions on fertility [1]. These complex relationships pose a challenge for accurately predicting future birth rates, yet such forecasts are crucial for guiding policy decisions effectively. By anticipating fertility trends, policymakers can better address demographic shifts and tailor strategies to counter challenges, ultimately impacting national economies and fiscal stability. Expanding on earlier studies that revealed the sensitivity of fertility rates to economic and political changes [2,3], this paper stresses the need to account for uncertainty arising from international geopolitical risks in fertility projections. By quantifying this uncertainty, policymakers gain a more profound comprehension of potential future situations, assisting them in making well-informed choices in the face of inherent uncertainties. Furthermore, assessing the efficacy of various forecasting strategies yields significant observations on their advantages and disadvantages, which are crucial for choosing the most appropriate methods for given situations.

This research investigates a methodology for projecting fertility rates in Scotland using time series analysis. We use a forecasting framework to assess the efficacy of conventional linear models in comparison to non-linear machine learning techniques. This comparative study seeks to provide insight into the appropriateness and dependability of these methods in various demographic contexts. The current body of research provides several techniques for predicting fertility, including principal component analysis, functional data models, time series models, and Bayesian approaches [4,5,6,7,8,9]. Alkema et al. (2011) conducted a thorough examination of different forecasting methods, emphasizing the significant challenges posed by uncertainty in demographic projections [10]. De Iaco and Maggio (2016) delved deeper into this issue by utilizing ARIMA methods to predict fertility parameters in Italy, while Mazzuco and Scarpa (2011) concentrated on bimodal fertility patterns using a specialized distribution [11,12]. Although these studies offer valuable insights into the factors influencing fertility rates, the utilization of non-linear techniques in forecasting still lags, as highlighted by Makridakis et al. (2018) [13]. To our knowledge, this is the first study that investigates the predictive power of geopolitical risks in birth rate data, offering various insights for policymakers. By understanding the potential impact of geopolitical risks on demographic trends, policymakers can make more informed decisions regarding resource allocation, social services, and economic planning. This novel approach highlights the importance of considering geopolitical factors in demographic studies, paving the way for future research in this area. Furthermore, while past studies have provided valuable insights into the factors influencing fertility rates, the adoption of non-linear techniques in forecasting remains somewhat limited. Our research addresses this gap by examining the potential of machine learning tree-based algorithms and boosting techniques alongside traditional linear models. To sum up, this paper makes several key technical contributions to the field of demographic forecasting by integrating advanced machine learning techniques and classical statistical procedures to analyze birth rate statistics, with a specific focus on Scotland. First, this study employs a variety of machine learning algorithms, including non-linear models such as XGBoost, Random Forest, and Extra Trees, to predict birth rates. These models demonstrate superior predictive accuracy compared to traditional linear regression models, particularly in handling complex, non-linear relationships within the data. Second, by introducing the Geopolitical Risk Index as an additional predictor, this research highlights the significant impact of geopolitical concerns on birth rate forecasts. This approach provides a novel way to quantify and integrate geopolitical instability into demographic forecasting models, enhancing their predictive power. Third, this study conducts a comprehensive comparison between machine learning models and classical statistical procedures, showcasing the advantages and limitations of each approach. This comparison provides valuable insights into the effectiveness of different methodologies for forecasting birth rates under varying conditions. Fourth, this research delves into how geopolitical instability—such as conflict, political turmoil, and disruptions to healthcare services—affects birth rates. By examining these factors, this study provides a deeper understanding of the socio-political determinants of demographic trends, highlighting the need for incorporating such variables in predictive models. Fifth, by utilizing a rolling estimation window approach and cross-validation for hyperparameter tuning, this study ensures robust and reliable model performance. The experimentation with various window sizes and hyperparameter configurations adds to the methodological rigor, allowing for the identification of optimal forecasting parameters. Overall, this paper demonstrates the effectiveness of integrating machine learning techniques with traditional statistical methods to produce precise birth rate predictions. It underscores the importance of considering geopolitical risks in demographic forecasting, offering valuable insights for policymakers, healthcare providers, and researchers concerned with the dynamics of birth rates.

Our focus is on forecasting births in Scotland, a region exhibiting consistently lower fertility rates compared to England and Wales. This disparity is primarily attributed to lower childbearing rates among women in their thirties and forties. Given Scotland’s aging population, declining birth rate, and the evolving post-Brexit landscape (www.gov.scot), accurate birth forecasts are crucial for informing national strategies. We conduct an out-of-sample forecasting exercise, evaluating the performance of various regression approaches across different forecasting horizons and accuracy measures. In our forecasting exercise, we consider an additional predictor to forecast the number of births, namely the Geopolitical Risk Index. The correlation between fertility rates and geopolitical risk is a crucial factor in shaping the direction of societies, economies, and politics. Fertility rates, which measure the number of births in a certain population, are crucial demographic statistics that influence the composition and long-term viability of civilizations. Geopolitical risk, which includes a range of political, economic, and social uncertainties, has an impact on the stability of nations, cooperation across regions, and relations between countries. Geopolitical risk, which includes political, economic, and social factors, has a significant impact on socioeconomic indicators. Geopolitical tensions, wars, and instability have the potential to disrupt international commerce, investment flows, and economic policies, which may hinder economic growth and development. Elevated geopolitical risk often results in heightened uncertainty, which may discourage firms from making long-term investment choices, diminish consumer confidence, and contribute to market volatility. Furthermore, geopolitical occurrences such as military wars, trade disagreements, or penalties may have a direct influence on the accessibility of resources, the functioning of supply chains, and the pricing of commodities. As a result, these factors can affect inflation rates and indices that measure the cost of living. Furthermore, geopolitical instability may exacerbate social tensions, contribute to internal displacement, and amplify societal grievances, potentially leading to unrest, migration flows, and humanitarian crises. In summary, the interconnectedness of geopolitical dynamics with socioeconomic variables underscores the importance of comprehensively assessing and managing geopolitical risks for fostering sustainable economic and social development on both national and global scales [14,15]. In our context, we consider whether during periods of conflict or political unrest, individuals may delay or forego childbirth due to the fear of their own safety or the well-being of their children. Moreover, disruptions in healthcare services, including access to prenatal care and maternal health facilities, may impact birth outcomes and contribute to changes in birth rates. Additionally, geopolitical events can influence broader social and economic factors that indirectly affect fertility rates, such as employment opportunities, income levels, and government policies related to family planning and social welfare [16].

Considering all the above, we delve into the complex relationship between fertility rates and geopolitical risk, highlighting the multifaceted dimensions of this relationship. At the heart of our analysis lies the recognition of fertility rates as not merely demographic phenomena, but as dynamic variables interconnected with geopolitical realities. Goldstone (2002) highlights the role of demographic factors, including fertility rates, in shaping the vulnerability or resilience of nations to geopolitical shocks [17]. Overall, our results indicate that including Geopolitical Risk Index as a predictor can enhance the accuracy of birth rate forecasts by capturing the influence of political instability on fertility decisions.

The rest of this paper is structured as follows: In Section 2, we outline the data utilized in the out-of-sample forecasting exercise and detail the methodology employed. Section 3 delves into the results of the analysis. Lastly, Section 4 concludes this paper.

## 2. Methodology

This section presents the machine learning approaches used to forecast births in Scotland. The current body of work mostly focuses on fertility modeling rather than fertility forecasting, as thoroughly examined by Booth (2006) and Bohk-Ewald et al. (2018) [18,19]. However, recent attention has shifted towards machine learning methodologies in forecasting, demonstrating notable improvements in accuracy across various disciplines. Our analysis encompasses tree-based algorithms such as Random Forest and boosting algorithms, specifically Extreme Gradient Boosting and Light Gradient Boosting Machine. Furthermore, we include Linear Regression in a comparison exercise with the non-linear machine learning methodologies. Overall, these methods are employed to explore their efficacy in accurately predicting births through an out-of-sample forecasting exercise with the inclusion of geopolitical risk as an additional predictor.

### 2.1. Random Forest

Breiman (2001) proposed the Random Forest algorithm, an ensemble method leveraging multiple decision trees [20]. Decision trees recursively partition data based on feature values to create homogenous groups with respect to a target variable. Every tree in a Random Forest is formed individually by employing a bootstrap trial of the real data (D with n samples and m features) along with a subset of features that are random. In doing so, we reduce potential issues of correlation between trees, which in turn improves the overall model robustness and qualifies overfitting.

A decision tree (T) is structured as a sequence of nodes that branch out. At each node, a specific feature (j) and a threshold for division are selected to effectively divide the data into two subsets, aiming to reduce error metrics such as the mean squared error (MSE), particularly in regression tasks. This process of recursive partitioning persists until predefined termination conditions are satisfied, such as attaining a maximum depth or meeting a minimum threshold of samples per leaf node. In the context of regression challenges, the ultimate prediction commonly entails aggregating predictions from all trees within the forest.

### 2.2. Extra Trees

Geurts et al. (2006) [21] proposed the Extra Trees algorithm, which is a regression-based ensemble learning method that expands on the ideas of decision trees. Like Random Forest, it follows a top–down approach to construct a set of regression trees. However, Extra Trees combines unique attributes that determine the qualities of individual trees and the overall behavior of the ensemble. Primarily, Extra Trees diverges from Random Forest by using a random selection of split points instead of the painstaking selection process aimed at minimizing the prediction error at each node. The randomness in decision trees leads to weaker correlations between trees compared to those within a Random Forest ensemble [22,23,24]. Furthermore, Extra Trees diverges from Random Forest by utilizing the entire training dataset for tree construction, in contrast to Random Forest’s utilization of bootstrapped samples. This variance in data incorporation fosters heightened diversification among the trees that constitute the Extra Trees ensemble.

### 2.3. Extreme Gradient Boosting (XGBoost)

XGBoost (Chen and Guestrin, 2016) is an efficient and scalable tree boosting algorithm [25]. It falls under the umbrella of tree boosting machine learning methods and has gained significant recognition for its effectiveness in various machine learning and data mining tasks.

(1) $$F_{k}\left(x\right)=\sum_{k=1}^{K}f_{k}\left(x\right),\;f_{k}\left(x\right)\in F$$

XGBoost capitalizes on a function space, denoted as F, which encompasses the entirety of potential regression trees. Within this function space, the algorithm endeavors to approximate an unidentified function through a series of boosting iterations, denoted as K. In each iteration, a new Classification and Regression Tree (CART) is introduced to augment the ensemble.

(2) $$L=\sum_{i-1}^{n}\Psi (y_{i,}F_{k}\left(x_{i}\right))+\sum_{k=1}^{K}\Omega (f_{k})$$

To avoid overfitting, XGBoost takes on a regularized objective function. This function has a loss function (Ψ(*)) *that measures forecast error and a regularization term* (Ω(*)) that in turn penalizes the complexity of the model.

(3) $$\Omega_{\left(f\right)}=\gamma T+0.5*{\lambda \|\omega \|}^{2}$$

The regularization component relies on the L2 norm of leaf weights (||ωt||²), where *γ* represents a predetermined coefficient. This strategy, drawing inspiration from regularized greedy forests, seeks to mitigate the influence of individual trees, thereby enhancing the resilience of the ensemble. XGBoost employs gradient descent for optimizing the objective function, concentrating particularly on first-order gradient statistics to determine the most favorable model parameters.

### 2.4. Light Gradient Boosting Machine (LGBM)

The Light Gradient Boosting Machine (LGBM) is an ensemble learning technique that enhances the classic gradient boosting framework by gradually including weak decision trees to improve prediction accuracy. LGBM differentiates itself from conventional gradient boosting algorithms by including Gradient-One-Side Sampling (GOSS). This approach prioritizes data points that have significant changes in the loss function while constructing the tree. GOSS accelerates model convergence and shows potential for improving regression performance by giving priority to the most informative occurrences.

### 2.5. Evaluation Metrics

For the evaluation of regression model performance, researchers have employed diverse metrics [26,27]. In this study, we adopt three widely utilized criteria: Mean Absolute Error (MAE), Root Mean Square Error (RMSE), and Symmetric Mean Absolute Percentage Error (SMAPE).

The *MAE* metric is defined as follows:

(4) $$MAE=\frac{1}{n}\sum_{i=1}^{n}\left|X_{i}-Y_{i}\right|$$

where $X_{i}$ stands for the predicted values and $Y_{i}$ stands for the actual values. The *RMSE* metric can be given as follows:

(5) $$RMSE=\sqrt{\frac{1}{n}\sum_{i=1}^{n}{\left(X_{i}-Y_{i}\right)}^{2}}$$

Finally, the SMAPE metric is defined as follows:

(6) $$SMAPE=\frac{100\%}{n}\sum_{i=1}^{n}\frac{\left|X_{i}-Y_{i}\right|}{(\left|X_{i}\right|+Y_{i})/2}$$

## 3. Data

This research uses machine learning predictive models to analyze birth data in England, with a specific emphasis on Scotland. We use birth data sourced from www.nrscotland.gov.uk, which consists of authoritative country-level records of monthly births. The time frame under consideration is explicitly limited to the period between January 1998 and December 2022. In this study, we use a logarithmic modification of the monthly births’ variable. In addition, we perform a nonparametric unit root test to ascertain the stationarity of the variable. The findings suggest that the birth series variable may be used in its logarithmic form without any further modification.

Furthermore, we utilize Geopolitical Risk Index data to explore the predictive value of the corresponding index in forecasting models to predict births. The country-specific Geopolitical Risk (GPR) Indexes reflect automated text-search results obtained from electronic newspaper archives. We utilize GPR data obtained from https://www.matteoiacoviello.com/gpr.htm, accessed on 1 May 2024. For the purposes of our analysis, we rely on the use of the Caldara and Iacoviello (2022) Geopolitical Risk Indexes for 44 advanced and emerging countries that determine the monthly share of all newspaper articles from 1985 to the present that meet specific criteria for inclusion in the GPR index and mention the name of the country or its major cities [28]. Each index represents a monthly share of newspaper articles and provides insight into the U.S. perspective on risks associated with or involving the respective country. We focus on the sample period from January 1998 to December 2022, as in the births data sample. The descriptive statistics for monthly births variable and the Geopolitical Risk Index are presented in Table 1. The Jarque–Bera test is used to test the hypothesis of normality, and the *p*-value is reported below. 

**Figure 1:**
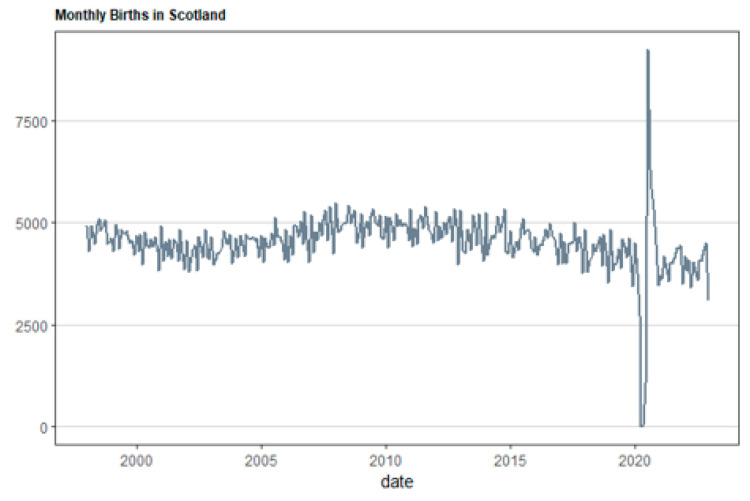
Total number of births in Scotland in months for the period from January 1998 to December 2022.

**Figure 2:**
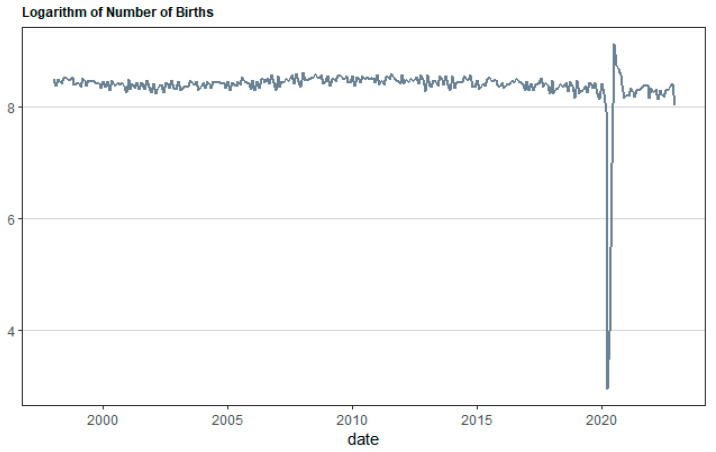
Logarithmic form of monthly births in Scotland for the period from January 1998 to December 2022.

**Figure 3:**
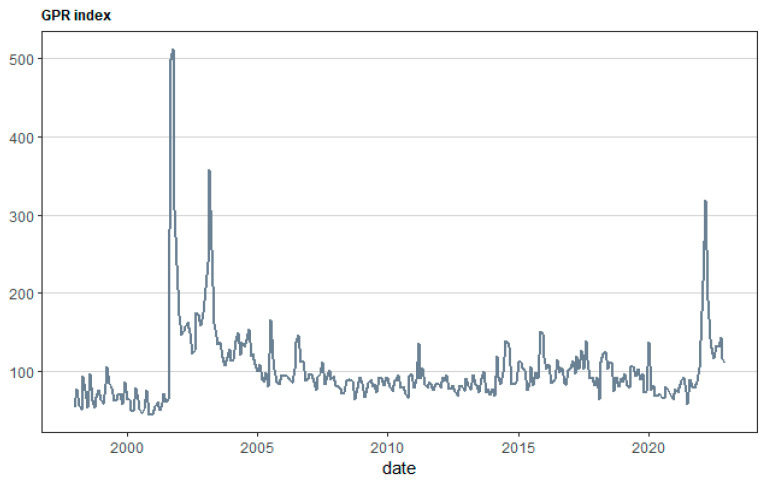
Geopolitical Risk Index for the sample period covering the period between January 1998 and December 2022.

**Figure 4:**
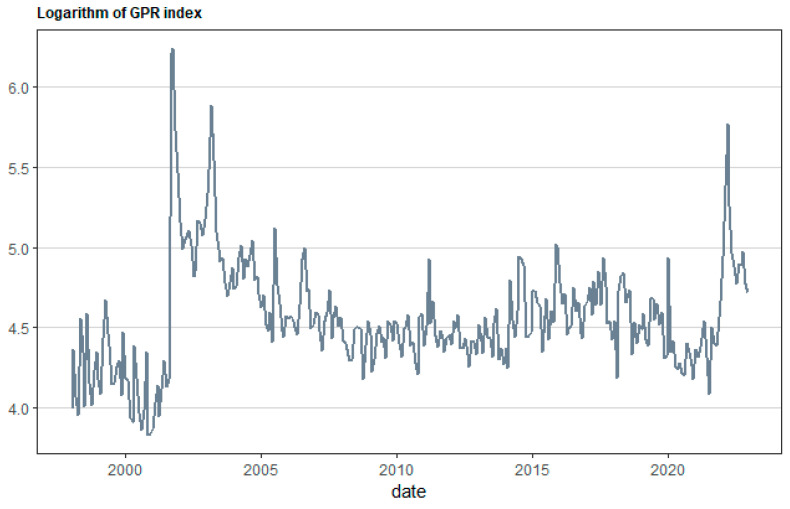
Logarithmic transformation of the Geopolitical Risk Index for the sample period covering the period between January 1998 and December 2022.

**Figure 5:**
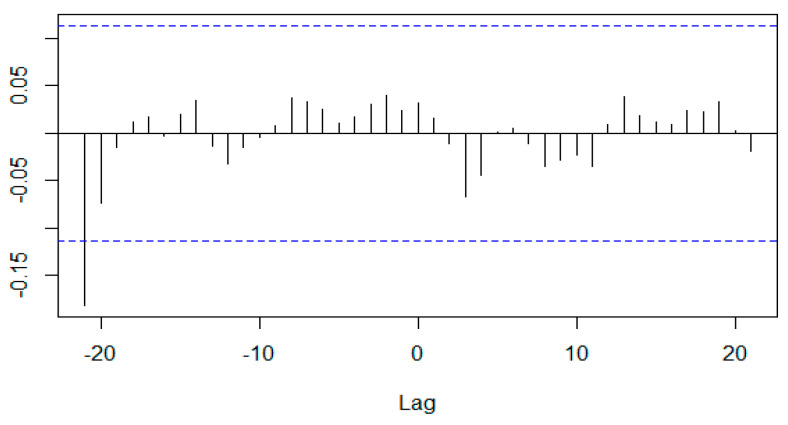
Cross-correlation plot between births and Geopolitical Risk Index using the logarithmic transformation of both variables.

Figure 1, Figure 2, Figure 3 and Figure 4 illustrate the overall monthly birth count in Scotland throughout the specified period. The data indicate a possible correlation between the COVID-19 epidemic and birth rates. The epidemic may have worsened pre-existing worries, namely economic ones, for young couples contemplating motherhood. Financial stability is a well-studied element that affects the choices made by individuals and couples when it comes to family planning. This emphasizes the need to implement a thorough national strategy to tackle the decreasing birth rate in Scotland. The suggested forecasting exercise has the potential to be a helpful tool for understanding demographic changes in Scotland and for guiding the creation of suitable policy interventions. Figure 5 displays the cross-correlation plot of the two variables under investigation. The cross-correlation graph indicates a possible lead–lag link between the series, with a lag of −20.

## 4. Results

In this paper, we investigate the application of machine learning for monthly birth rate prediction in Scotland. The declining birth rate and fertility rate highlight the need for more effective forecasting methods. The results of our forecasting experiment can offer valuable insights for policymakers, healthcare providers, and stakeholders concerned with demographic trends and future planning.

In this section, the results of the out-of-sample forecasting exercise using various machine learning regression methods are presented. In our analysis, a rolling estimation window approach with 24 observations was considered [29,30,31,32,33]. Like this approach, Tsay (2014) emphasized the importance of data splitting into training and testing sets, typically using an 80/20 ratio, to enhance model validation and reliability [30]. The method of hyperparameter tuning and lag selection via cross-validation, as employed in this study, is consistent with the recommendations of Bergmeir et al. (2018), who highlighted cross-validation’s critical role in optimizing time series forecasting models [31]. Moreover, the exploration of different window sizes and hyperparameter configurations, such as learning rates and the number of estimators, reflects the strategies outlined by Goodfellow, Bengio, and Courville (2016) for improving machine learning model performance [32]. The use of specific R packages like timetk, tidymodels, random Forest, and lubridate is supported by past studies, such as Kuhn and Johnson (2013), which underscore the utility of these tools in implementing robust and efficient machine learning workflows for time series analysis [33]. The data were split into training and testing sets using an 80/20 ratio. Hyperparameter tuning for each machine learning model and lag selection for the birth variable and the GPR Index variable were performed via cross-validation. Window sizes of 1, 3, 6, 9, 12, and 20 months were explored, with 20 months ultimately chosen as the optimal value. Additionally, different hyperparameter configurations were tested for each model, including learning rates (0.00001, 0.00005, 0.0001, 0.0005, and 0.001) and the number of estimators (50, 100, and 500). The analysis was implemented using the timetk, tidymodels, randomForest, and lubridate packages in R.

We employed three common metrics (RMSE, MAE, and SMAPE) to assess the predictive performance of various forecasting models for each volatility estimator and forecasting horizon [34,35]. Additionally, we utilized the Model Confidence Set (MCS) approach by Hansen et al. (2011) to identify statistically superior models [36]. MCS iteratively removes models with demonstrably lower accuracy, allowing for robust comparisons.

The corresponding Table 2 and Table 3 present the evaluation results. Each Table summarizes the metrics for all machine learning models considered. For instance, Table 2 displays the results for monthly univariate out-of-sample birth rate prediction. The results reveal slight variations in RMSE, MAE, and SMAPE across the models. Notably, Random Forest and Extra Trees exhibited the lowest metric values for the 1-month horizon, suggesting strong performance. Boosting algorithms (XGBoost and LightGBM) also demonstrated promising results with lower metric values. Interestingly, Linear Regression yielded the worst results across all horizons. However, it is important to note that the observed differences in metric scores were generally small. Additionally, results for the 6-months ahead forecasting horizon (Table 2, panel B) indicate that the proposed bagging tree-based models appear very competitive in the multi-step ahead forecasting setting.

Table 3 (Panels A and B) presents the results for the 1-month ahead and 6-months ahead forecasting horizons, respectively, with the inclusion of the Geopolitical Risk Index as an additional predictor [7,19,37,38]. The results are similar in this case, as well as in the regression models. However, we observed a slight improvement in the forecasting ability of the regression models, as observed in the slightly lower metric values. We find that the proposed bagging tree-based models are particularly effective for 6-months ahead forecasting horizon. Additionally, the LightGBM algorithm exhibited promising results. Overall, our results indicate that non-linear machine learning regression models, particularly Random Forest, Extra Trees, and XGBoost, exhibit promising capabilities in forecasting the future number of births in Scotland, while the inclusion of Geopolitical Risk Index as an additional predictor adds predictive value to the corresponding models to predict the number of births. However, slight differences in the metric values were observed across the models and forecasting horizons considered. These findings align with and extend those of other academic studies that focused on birth rate forecasting. For instance, a study by Bohk and Rau (2014) on fertility forecasting emphasized the importance of incorporating external variables, such as economic indicators, to improve predictive accuracy [39].

The inclusion of GPR in this study similarly enhances the model’s performance, indicating that geopolitical factors can provide valuable predictive information, much like economic variables. The effectiveness of bagging tree-based models, particularly for the 6-months ahead forecasting horizon, aligns with research by Kunz, Schmertmann, and Holzmann (2019), who found that ensemble methods like Random Forest are robust in capturing the complexities and non-linearities inherent in demographic data [7]. Their study demonstrated that tree-based models often outperform traditional linear models in forecasting birth rates due to their ability to handle non-linear relationships and interactions within the data. The promising results of the LightGBM algorithm in this study are consistent with findings from Bojer and Meldgaard (2021), who highlighted the efficiency and accuracy of gradient boosting algorithms in various forecasting tasks, including demographic predictions [37]. Their research supports the notion that LightGBM, with its advanced boosting techniques and ability to manage large datasets, is well-suited for complex forecasting scenarios.

Furthermore, the slight differences in metric values across models and forecasting horizons observed in this study are comparable to the variability reported by Schmertmann et al. (2014), who noted that model performance can vary significantly depending on the forecast horizon and the specific characteristics of the birth rate data being analyzed [38]. This highlights the importance of selecting appropriate models and tuning parameters based on the specific forecasting context. Overall, the results of this study reinforce the findings of previous research by demonstrating the effectiveness of non-linear machine learning models, such as Random Forest, Extra Trees, XGBoost, and LightGBM, in forecasting birth rates. The inclusion of the Geopolitical Risk Index as an additional predictor further enhances the models’ predictive capabilities, providing a more comprehensive approach to demographic forecasting.

## 5. Discussion and Conclusions

This study examines the effectiveness of several machine learning regression models in accurately predicting the number of births in Scotland using data that is not included in the model training process. The results suggest that non-linear models, namely XGBoost and tree-based algorithms with bagging (Random Forest and Extra Trees), show promising outcomes in predicting births. In contrast, Linear Regression consistently produced less precise forecasts for both periods. In addition, the inclusion of the Geopolitical Risk Index as an extra factor in the forecasting models improved their ability to make accurate predictions.

Geopolitical instability has a substantial influence on birth rates and fertility rates. War, civil unrest, and political uncertainty can create economic hardship and feelings of insecurity that further affect people’s decisions to have children. Moreover, relocation caused by violence may alter family dynamics and impede access to healthcare, thereby exacerbating the challenges of childbirth. The outcomes of our predictive trial may provide significant perspectives for politicians, healthcare professionals, and others interested in demographic patterns and future strategizing. The findings of this research provide several interesting perspectives. Tree-based algorithms and bagging approaches, which are non-linear machine learning models, are especially successful for predicting birth rates. The significant improvement in forecast precision by using the Geopolitical Risk Index underscores the crucial significance of geopolitical stability in demographic patterns. Policymakers, healthcare professionals, and stakeholders may use these observations to obtain a deeper understanding and predict fluctuations in birth rates, thereby facilitating more knowledgeable decision making and strategic planning. This study highlights the significance of integrating a wide range of factors and using sophisticated machine learning methods in demographic forecasting.

Although the findings show promise, this research is subject to significant constraints. The scope of this study is confined to Scotland, hence potentially limiting the applicability of the results to other areas or nations with distinct socioeconomic and geopolitical circumstances. Furthermore, the forecasting models were trained and evaluated using historical data, which could not completely include future patterns caused by unforeseen geopolitical events or changes in socioeconomic circumstances. In addition, this research specifically examined a restricted range of machine learning algorithms and hyperparameters, which may have hampered the ability to uncover more efficient models or configurations.

Subsequent investigations should contemplate broadening the geographic range of the study to include other nations or areas in order to authenticate the models’ applicability. Additionally, it would be advantageous to investigate a wider array of machine learning methods and perform more thorough hyperparameter tuning in order to determine the most optimum configurations. In addition, including supplementary predictors, such as economic data, healthcare access measurements, and social policies, might provide a more thorough comprehension of the variables that impact birth rates. Incorporating real-time data into longitudinal research might improve the models’ capacity to adjust to changing geopolitical and socioeconomic settings.

## Figures and Tables

**Table 1:** Summary statistics for births in Scotland and the Geopolitical Risk Index.

Descriptive Statistics	Births	GPR Index
Mean	8.381	4.563
Median	8.427	4.512
Maximum	9.131	6.241
Minimum	2.944	3.830
std. Dev.	0.429	0.349
Skewness	−10.526	1.299
Kurtosis	124.054	6.928
Jarque–Bera	18,8717 ***	277.36 ***
p-value	[0.000]	[0.000]

**Note**: In this table the descriptive statistics for monthly births variable and the Geopolitical Risk Index are presented. The Jarque–Bera test is used to test the hypothesis of normality, and the *p*-value is reported below. *** indicate statistical significance at a 1% level.

**Table 2:** Estimation results for the births in Scotland (univariate one-step-ahead and multi-step-ahead out-of-sample).

**Panel A**	**H = 1**		
**Model**	**MAE**	**RMSE**	**SMAPE**
LightGBM	0.95	1.28	0.48
Extra Trees	0.91	1.24 *	0.46
Random Forest	0.91	1.25	0.45
Extreme Gradient Boosting	0.94	1.27 *	0.47
Linear Regression	1.08	1.38	0.52
**Panel B**	**H = 6**		
**Model**	**MAE**	**RMSE**	**SMAPE**
LightGBM	1.06	1.38	0.53
Extra Trees	1.05	1.37 *	0.52
Random Forest	1.05	1.38	0.51
Extreme Gradient Boosting	1.04	1.36 *	0.51
Linear Regression	1.07	1.40	0.53

**Note:** This table reports the out-of-sample results for predicting births in Scotland (H = 1 and 6 months). (*) indicates models that are included in the MCS at the 1% significance level.

**Table 3:** Estimation results for the births in Scotland, including the Geopolitical Risk Index (one-step-ahead and multi-step-ahead out-of-sample).

**Panel A**	**H = 1**		
**Model**	**MAE**	**RMSE**	**SMAPE**
LightGBM	0.33	0.43	0.55
Extra Trees	0.32	0.42 *	0.54
Random Forest	0.31	0.41 *	0.53
Extreme Gradient Boosting	0.33	0.42 *	0.54
Linear Regression	0.45	0.52	0.67
**Panel B**	**H = 6**		
**Model**	**MAE**	**RMSE**	**SMAPE**
LightGBM	0.40	0.48	0.63
Extra Trees	0.38	0.47 *	0.62
Random Forest	0.39	0.48 *	0.61
Extreme Gradient Boosting	0.37	0.46 *	0.61
Linear Regression	0.46	0.53	0.67

**Note:** This table reports the out-of-sample results for predicting births in Scotland with the inclusion of Geopolitical Risk Index (H = 1 and 6 months). (*) indicates models that are included in the MCS at the 1% significance level.

## Data Availability

Data available on request due to restrictions. The data presented in this study are available on request from the corresponding author.
